# Inositol for Polycystic Ovary Syndrome: A Systematic Review and Meta-analysis to Inform the 2023 Update of the International Evidence-based PCOS Guidelines

**DOI:** 10.1210/clinem/dgad762

**Published:** 2024-01-02

**Authors:** Victoria Fitz, Sandro Graca, Shruthi Mahalingaiah, Jing Liu, Lily Lai, Ali Butt, Mike Armour, Vibhuti Rao, Dhevaksha Naidoo, Alison Maunder, Guoyan Yang, Vaishnavi Vaddiparthi, Selma F Witchel, Alexia Pena, Poli Mara Spritzer, Rong Li, Chau Tay, Aya Mousa, Helena Teede, Carolyn Ee

**Affiliations:** Division of Reproductive Endocrinology and Infertility, Department of Obstetrics and Gynecology, Massachusetts General Hospital, Boston, MA 02114, USA; School of Health and Society, Faculty of Education, Health and Wellbeing, University of Wolverhampton, Wolverhampton WV1 1LY, UK; Division of Reproductive Endocrinology and Infertility, Department of Obstetrics and Gynecology, Massachusetts General Hospital, Boston, MA 02114, USA; NICM Health Research Institute, Western Sydney University, Penrith 2751, Australia; Primary Care Research Centre, University of Southampton, Southampton SO17 1BJ, UK; NICM Health Research Institute, Western Sydney University, Penrith 2751, Australia; NICM Health Research Institute, Western Sydney University, Penrith 2751, Australia; NICM Health Research Institute, Western Sydney University, Penrith 2751, Australia; NICM Health Research Institute, Western Sydney University, Penrith 2751, Australia; NICM Health Research Institute, Western Sydney University, Penrith 2751, Australia; NICM Health Research Institute, Western Sydney University, Penrith 2751, Australia; NICM Health Research Institute, Western Sydney University, Penrith 2751, Australia; UPMC Children's Hospital of Pittsburgh, University of Pittsburgh, Pittsburgh, PA 15260, USA; Discipline of Paediatrics, The University of Adelaide and Robinson Research Institute, Adelaide 5005, Australia; Gynecological Endocrinology Unit, Division of Endocrinology, Hospital de Clinicas de Porto Alegre; Department of Physiology, Universidade Federal do Rio Grande do Sul, Rio Grande do Sul 91509-900, Brazil; Department of OB & GYN, Reproductive Medical Center, Peking University Third Hospital, Beijing 100191, China; Monash Centre for Health Research and Implementation, Monash University, Clayton 3800, Australia; Monash Centre for Health Research and Implementation, Monash University, Clayton 3800, Australia; Monash Centre for Health Research and Implementation, Monash University, Clayton 3800, Australia; NICM Health Research Institute, Western Sydney University, Penrith 2751, Australia; Caring Futures Institute, Flinders University, Bedford Park 5042, Australia

**Keywords:** inositol, polycystic ovary syndrome, nutrients

## Abstract

**Context:**

Insulin resistance is common in women with polycystic ovary syndrome (PCOS). Inositol may have insulin sensitizing effects; however, its efficacy in the management of PCOS remains indeterminate.

**Objective:**

To inform the 2023 international evidence-based guidelines in PCOS, this systematic review and meta-analysis evaluated the efficacy of inositol, alone or in combination with other therapies, in the management of PCOS.

**Data Sources:**

Medline, PsycInfo, EMBASE, All EBM, and CINAHL from inception until August 2022.

**Study Selection:**

Thirty trials (n = 2230; 1093 intervention, 1137 control), with 19 pooled in meta-analyses were included.

**Data Extraction:**

Data were extracted for hormonal, metabolic, lipids, psychological, anthropometric, reproductive outcomes, and adverse effects by 1 reviewer, independently verified by a second.

**Data Synthesis:**

Thirteen comparisons were assessed, with 3 in meta-analyses. Evidence suggests benefits for myo-inositol or D-chiro-inositol (DCI) for some metabolic measures and potential benefits from DCI for ovulation, but inositol may have no effect on other outcomes. Metformin may improve waist-hip ratio and hirsutism compared to inositol, but there is likely no difference for reproductive outcomes, and the evidence is very uncertain for body mass indexI. Myo-inositol likely causes fewer gastrointestinal adverse events compared with metformin; however, these are typically mild and self-limited.

**Conclusion:**

The evidence supporting the use of inositol in the management of PCOS is limited and inconclusive. Clinicians and their patients should consider the uncertainty of the evidence together with individual values and preferences when engaging in shared decision-making regarding the use of inositol for PCOS.

Polycystic ovary syndrome (PCOS) is a common metabolic and reproductive condition affecting females of reproductive age (1). PCOS is characterized by hyperandrogenism, ovulatory dysfunction, and/or polycystic ovarian morphology (2). Obesity is more common in women with PCOS compared to women without (3), and women with PCOS are more likely to be affected by insulin resistance irrespective of body mass index (BMI) (4).

Metformin, a biguanide that decreases hepatic glucose production and increases body insulin sensitivity, is recommended in the 2018 international evidence-based PCOS guideline (5) for management of anthropometric and metabolic outcomes in PCOS. However, other options to treat insulin resistance may be desired by women with PCOS due to gastrointestinal side effects related to metformin. Hence, there is a need for alternatives to metformin for managing insulin resistance in PCOS.

Inositol was originally isolated from muscle cells in 1850 by Johann Joseph Scherer (6). Inositols are structural components of cell membranes (eg, phosphatidyl-inositol phosphate lipids) and participate in hormone signal transduction (eg, inositol triphosphate). There are 9 stereo-isomers of inositol of which myo-inositol (MI) is the most abundant in the human body (7). In humans, dietary intake and endogenous synthesis of MI occurs.

Mechanistically, MI promotes translocation of the glucose transporter type 4 to the plasma membrane for glucose uptake (7) and also reduces release of free fatty acids from adipose tissue (8). MI is also involved in reproductive functions, including follicle stimulating hormone (FSH)-mediated pathways, which regulate the proliferation and maturation of granulosa cells (9). Insulin stimulates unidirectional conversion of MI to D-chiro-inositol (DCI) (10), which stimulates glycogen production and facilitates additional uptake of glucose through mobilization of glucose transporter type 4 transporters (11). It has been hypothesized that overproduction of insulin in PCOS enhances MI to DCI conversion, which results in an increased DCI and decreased MI concentration in follicular fluid (12). In women without PCOS, the ovarian MI:DCI ratio is 100:1, but in women with PCOS, the ratio is 0.2:1 (13). MI is also postulated to enhance aromatase synthesis in granulosa cells and therefore reduce androgen production (8). It has been suggested that a 40:1 ratio of MI:DCI is physiological, and provision of inositol in this ratio has reverted PCOS phenotypes in mouse models (14).

In view of the potential benefits of inositol, thorough and critical evaluation of its efficacy in the treatment of PCOS is essential. The aim of this systematic review and meta-analysis was to evaluate the effectiveness of inositol alone or in combination with other therapies for the management of hormonal and clinical PCOS features, weight, and reproductive outcomes.

## Materials and Methods

This systematic review and meta-analysis was conducted to inform the 2023 update of the International Evidence-based Guideline on the Assessment and Management of PCOS (15) and followed the Preferred Reporting Items for Systematic Reviews and Meta-Analyses guidelines (16). The protocol was prospectively registered with PROSPERO (CRD 42022356057). The review informed the guideline recommendation for the question “In adolescents and adults with PCOS, is inositol alone or in combination with other therapies effective for management of hormonal and clinical PCOS features, weight, and reproductive outcomes?” The PROSPERO registration includes the reproductive outcomes of interest; the other outcomes were inadvertently left off the registration. However, all the outcomes including hormonal, clinical, anthropometric, and reproductive were determined a priori by the guideline evidence synthesis team.

### Information Sources

We searched the following databases from inception until August 5, 2022: Medline (OVID), PsycInfo (EBSCO), EMBASE (OVID), All EBM (OVID), and CINAHL (EBSCO). A hand-search of reference lists of relevant recent systematic reviews was conducted to identify any trials that were not captured in our search. We asked experts in the field to identify any trials that were not identified in our search.

### Search Strategy

The search strategy and selection criteria were developed by the guideline evidence and key contact team, comprising an international team of evidence synthesis experts, reproductive endocrinologists, endocrinologists, pediatricians, and a general practitioner (family physician). The search strategy included terms for PCOS, inositol, and randomized controlled trials and is included in the supplemental data (17).

Only randomized controlled trials (RCTs) were included with an a priori defined Participant-Intervention-Comparison-Outcome framework, using the following criteria: (1) participants: females diagnosed with PCOS by Rotterdam, National Institutes of Health, or Androgen Excess and PCOS Society (AEPCOS) criteria of any age, ethnicity and weight; (2) intervention: inositol (MI or DCI alone or in combination), alone or combined with usual care (as defined by study authors), lifestyle, or any other interventions of any dose or duration; (3) comparator: placebo, usual care alone, lifestyle alone, or any other interventions (listed in intervention) or combinations of those listed in intervention. Where comparisons or co-interventions were used in 2 or more study groups, these should be the same across all groups, with inositol being the main difference between groups in order to isolate the effects of inositol. The only exception was that comparisons of inositol + folic acid (FA) vs another comparator (eg, placebo) was deemed to be eligible as the effect of FA on outcomes was thought to be minimal; (4) outcomes: hormonal, metabolic, lipids, psychological, anthropometric or reproductive outcomes, and adverse events [see supplementary data (17) for a full list of eligible outcomes].

### Selection Process and Data Extraction

Citations were imported into Covidence (18), where duplicates were removed in Covidence. Titles and abstracts and then full-text manuscripts were screened independently in duplicate by 3 reviewers (V.F., C.E., S.M.) independently using Covidence, and disagreements were resolved by discussion, with a third reviewer to adjudicate if needed. Data were extracted by 1 reviewer (A.B., C.E., D.N., J.L., L.L., S.G., V.F., V.R., V.V.) on study characteristics, participant characteristics at baseline, intervention, and outcomes using a data extraction template created by the guideline evidence team. Extracted data were then verified by a second reviewer (A.B., C.E., G.Y., L.L., M.A., S.M., V.F., V.R.).

### Integrity Assessment

Trial integrity was assessed by an integrity committee following the Research Integrity in Guideline Development (RIGID) framework developed by Mousa et al (2023; unpublished), as detailed in Section 6.7 of the guideline technical report (19). Here, studies were assessed using the Trustworthiness in Randomised Controlled Trials checklist (20), an integrity assessment tool similar to the Cochrane Research Integrity Assessment tool (21), which assesses studies on multiple domains related to integrity. Following the steps of the RIGID framework, studies were classified as low, moderate, or high risk for integrity concerns. Low-risk studies were included, while authors for moderate- and high-risk studies were contacted to clarify integrity concerns. Where a satisfactory response was received, those studies were subsequently “included.” Studies with no response were “not included,” while studies requiring additional time to provide the necessary information (eg, raw data, ethics protocols, etc.) are “awaiting classification” and have not been included in the review or analysis at this stage.

### Quality Appraisal

We assessed risk of bias for each trial using the Cochrane Risk of Bias 1.0 tool (22). Two reviewers independently assessed random sequence generation (selection bias), allocation concealment (selection bias), blinding of participants and personnel (performance bias), blinding of outcome assessment (detection bias), incomplete outcome data (attrition bias), selective reporting (reporting bias), and other biases. Any disagreements were resolved by discussion.

The certainty of evidence was assessed by M.A. and V.F. using the Grading of Recommendations, Assessment, Development and Evaluations (GRADE) approach, as outlined in the GRADE handbook (23). The GRADE approach uses 5 considerations (risk of bias, inconsistency of effect, indirectness, imprecision, and other bias including publication bias). The evidence can be downgraded from “high quality” by 1 level for serious (or by 2 levels for very serious) limitations, depending on assessments of these domains.

### Data Analysis

The key contacts (clinical leads) for the guideline judged the following outcomes as critical (for GRADE purposes): free testosterone (FT), homeostatic model assessment of insulin resistance (HOMA-IR), 2-hour glucose, BMI. The remaining outcomes were judged as important but not critical. Outcome data were extracted from original intention-to-treat results wherever possible or from per-protocol results if these were the only outcomes available. Heterogeneity was assessed using the *I^2^* statistic. Outcomes from individual studies were pooled using random effects models. For trials that used the same assessment method and provided continuous data, we reported mean difference (MD), converting units of measurement to standard units where required. Categorical outcomes were reported as odds ratios (OR). All pooled analyses were reported with 95% confidence intervals (CIs). We used RevMan (24) for statistical analysis. Publication bias was assessed by visual inspection of funnel plot asymmetry, where applicable. Subgroup analyses for menopausal stage, adolescents/adults, and BMI category were planned but were not conducted due to the small number of trials.

### Changes Since Protocol Was Registered

Anthropometric, hormonal, psychological, and metabolic outcomes were collected as determined a priori by the research team but inadvertently not included in the PROSPERO registration. We conducted a sensitivity analysis according to risk of bias, where only the studies that were assessed as low risk of selection bias and not at high risk of performance bias were included. We conducted supplementary analyses for critical outcomes only where we also included data from studies that were not included due to moderate-high risk of integrity concerns. We conducted supplementary analyses using standardized mean differences (SMD) for endocrinological outcomes where outcomes were collected at different laboratories and therefore potentially using different assays (see Supplementary Data S6).

## Results

**Figure 1.**
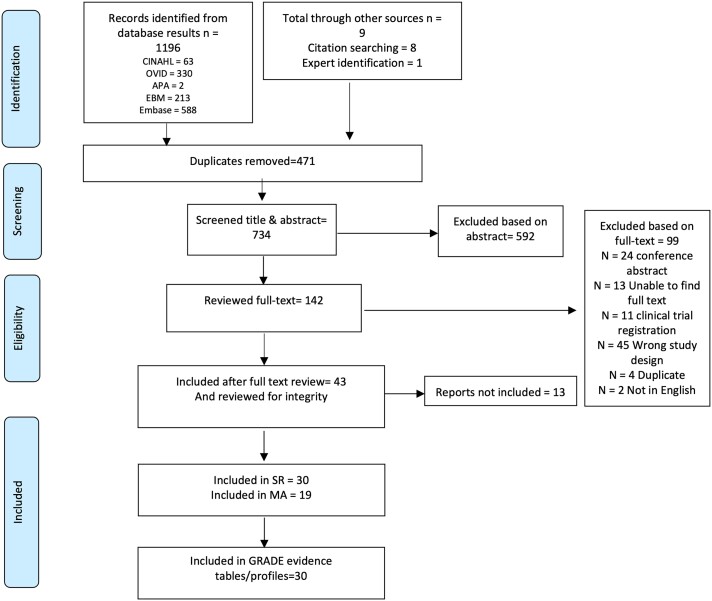
PRISMA flowchart of study selection. Abbreviations: PRISMA, Preferred Reporting Items for Systematic Reviews and Meta-Analyses.

See Fig. 1 for the Preferred Reporting Items for Systematic Reviews and Meta-Analyses flow chart of study selection. A total of 1535 citations were identified, of which 1534 were identified through database searches and 1 through expert identification, with 734 remaining after duplicates were removed. After title and abstract screening, an additional 592 were excluded, and 142 full-text manuscripts were reviewed for eligibility. Ninety-nine were excluded based on full text review, which left 43 trials then assessed for integrity. Thirty were assessed as low risk and included. Thirteen were assessed to be of moderate risk and were not included. (see supplementary data and Supplementary Table S1 for characteristics of moderate risk studies) (17). Table 1 provides details of study characteristics of the included studies. In total, there were 31 articles representing 30 unique trials and 2230 participants. There was 1 study including only adolescents (n = 106) (37). Fifteen trials were conducted in Italy (27, 29, 31-34, 38, 39, 44, 45, 47, 48, 50, 54, 55), 6 in India (26, 28, 30, 36, 41, 42, 51), 2 in Iran (25, 49), 2 in Venezuela (35, 43), and 1 each in Pakistan (37), Turkey (46), Spain (40), Denmark (52), and Bosnia and Herzegovina (53). Sample sizes for arms relevant for this study ranged from 8 to 195 with a mean sample size of 36 participants. Nineteen studies contributed to meta-analyses.

**Table 1.** Characteristics of included studies

Author, year, country, study design	Population/Setting	Sample size per group, age, BMI	Intervention details	Comparison/control details	Co-interventions	Outcomes, follow-up duration
Akbari Sene 2019 (25) Iran RCT	Women with PCOS (Rotterdam), with infertility who presented to an IVF center in Tehran, Iran	MI + FA = 25 Age = 31.3 ± 4.1 years BMI = 25.26 ± 5.2 kg/m^2^ FA alone = 25 Age = 29.78 ± 4.5 years BMI = 26.2 ± 4.52 kg/m^2^	4 g MI + 400 mcg FA daily	400 mcg FA daily	IVF done after treatment period of 1 month	Reproductive: embryo quality, rate of mature oocytes, rate of fertilization, relative gene expression levels in mural granulosa cells, cumulative pregnancy rate Follow-up: Until IVF outcome
Angik 2017 (26) India Parallel RCT	Women with PCOS (Rotterdam) Outpatient Obstetrics and Gynaecology Department in Tertiary Healthcare Centre, India	MI = 50 BMI = 24.10 ± 4.13 kg/m^2^ Metformin = 50 BMI = 23.23 ± 2.65 kg/m^2^ Age (total, n = 100) between 15 and 40 years	2 g MI daily for 6 months	1 g metformin daily for 6 months	Not reported	Androgenicity: mFG, testosterone Metabolic: fasting glucose, fasting insulin, HOMA-IR Anthropometric: BMI, waist circumference, WHR Reproductive: menstrual regularity, pregnancy rate Adverse events. Follow-up: 6 months
Artini 2013 (27) Italy Parallel RCT (not clear if blinded)	Women with PCOS presenting for planned IVF treatment at a university-based fertility clinic in Italy	MI + FA = 25 Age = 34.9 ± 2.1 years BMI = 26.5 ± 6.1 kg/m^2^ FA alone = 25 Age = 36.2 ± 2.3 years BMI = 23.3 ± 6.8 kg/m^2^	2 g MI + 400 mcg FA daily for 12 weeks prior to IVF	400 mcg FA	IVF after completing 12 weeks of placebo or intervention	Androgenicity: testosterone, androstenedione Metabolic: Glucose/insulin ratio, HOMA-IR, AUC insulin (2 hours after 75 g OGTT), 30 minutes and 60 minutes 75 g OGTT, insulin (fasting) Anthropometric: BMI IVF outcomes: oocytes, top quality oocytes, fertilization rate, top quality embryos, hCG positive, clinical pregnancy (pregnancy seen on US with + FHR), delivery rate Follow up: 12 weeks
Bahadur 2021 (28) India Parallel nonblinded RCT	Newly diagnosed women with PCOS (Rotterdam) Obstetrics and gynaecology department at medical institution	MI + DCI + metformin = 36 Age = 23.78 ± 4.46 years BMI = 25.29 ± 4.13 kg/m^2^ Metformin only = 36 Age = 21.89 ± 4.23 years BMI = 23.43 ± 4.75 kg/m^2^	1100 mg MI + 300 mg DCI + 1000 mg metformin, daily	1000 mg metformin, daily	None reported	Androgenicity: mFG score, total testosterone, DHEAS Metabolic: HOMA-IR index, fasting glucose, fasting insulin Lipids and other biomarkers: total cholesterol, LDL, HDL, triglycerides Anthropometric: waist circumference, waist-hip ratio, BMI Reproductive: menstrual cyclicity Follow-up: 6 months
Benelli 2016 (29) Italy RCT	Women with PCOS (Rotterdam) Department of Clinical and Experimental Medicine, University of Pisa, Italy	MI + DCI + FA = 21 Age = 23 ± 6.8 years BMI = 32 ± 4.8 kg/m^2^ FA only = 25 Age = 25 ± 7.3 years BMI = 31 ± 4.6 kg/m^2^	MI + DCI combined treatment at the ratio of 40:1 in soft gel capsule containing 550 mg of MI, 13.8 mg of DCI + 200 μg of FA (INOFOLIC® COMBI, Lo.Li.Pharma) 2 × day for 6 months	200 μg of FA (INOFOLIC® COMBI, Lo.Li.Pharma) 2 × day for 6 months	None reported	Androgenicity: free testosterone, SHBG, DHEAS, androstenedione Metabolic: fasting glucose, fasting insulin, HOMA-IR Follow-up: 6 months
Chirania 2017 (30) India Parallel 3-armed RCT (described as equivalence trial)	Women with PCOS (Rotterdam) Outpatient obstetrics and gynaecology dept in a medical college in India	MI = 26 Age = 23.92 ± 3.70 years BMI = 24.63 ± 3.32 kg/m^2^ MI + Metformin = 22 Age = 21.9 ± 3.45 years BMI 25.02 ± 9.14 kg/m^2^ Metformin = 28 Age = 23.68 ± 4.23 years BMI = 25.44 ± 2.68 kg/m^2^	MI only = 1 g MI daily for 4 months MI + Met = 1 g MI + 1 g metformin daily for 4 months	1 g metformin daily for 4 months	Not reported	Metabolic: fasting insulin Anthropometric: BMI, WHR Reproductive: menstrual regularity, pregnancy rate Follow-up: 4 months
Costantino 2009 (31) Italy Parallel double-blind RCT	Women with PCOS Setting not described	MI + FA = 23 Age = 28.8 ± 1.5 years BMI = 22.8 ± 0.3 kg/m^2^ FA alone = 19 Age = 27.1 ± 1.4 years BMI = 22.5 ± 0.3 kg/m^2^	4 g MI + 400 mcg FA daily	400 mcg FA daily	None	Androgenicity: total testosterone, free testosterone, androstenedione, DHEAS, SHBG Metabolic: fasting glucose, fasting insulin, AUC glucose, AUC insulin, whole-body insulin sensitivity (ISI comp) Lipids and other biomarkers: total cholesterol, triglycerides Anthropometric: BMI, WHR Follow-up: 6 weeks
Dona 2012 (32) Italy Parallel placebo-controlled RCT	Women with lean PCOS (Rotterdam) Department of Medical and Surgical Sciences at a university in Italy	MI = 18 Age = 23.5 ± 2.1 years BMI = 21.6 ± 1.9 kg/m^2^ Placebo = 8 Age = 23.6 ± 1.4 years BMI = 21.9 ± 0.6 kg/m^2^	1.2 g MI daily for 12 weeks	Matched placebo powder for 12 weeks	Subjects were instructed not to change their eating habits, activity level, or lifestyle during the study.	Androgenicity: total testosterone, androstenedione Metabolic: fasting glucose, fasting insulin, HOMA-IR, AUC glucose, AUC insulin Anthropometric: weight, BMI Follow-up: 3 months
Fruzzetti 2017 (33) Italy RCT (third arm of patients without PCOS not included)	Women with PCOS (Rotterdam) outpatient clinic of reproductive endocrinology of the University of Pisa, Italy, for oligo-amenorrhoea and clinical signs of hyperandrogenism	MI = 24 Age = 21.6 ± 6.6 years BMI = 27.3 ± 4.5 kg/m^2^ Metformin = 22 Age = 22.3 ± 6.0 years BMI = 28.4 ± 5.2 kg/m^2^	4 g MI + 400 mcg FA daily	1500 mg metformin daily		Androgenicity: hirsutism, acne Metabolic: HOMA-IR, AUC insulin, Matsuda index Anthropometric: BMI Reproductive: menstrual cycle Adverse events Follow-up: 6 months
Genazzani 2008 (34) Italy RCT	Women with PCOS (Rotterdam) Gynaecological Endocrinology Centre at the University of Modena	MI + FA = 10 BMI = 29 ± 1.6 kg/m^2^ (SEM) FA alone = 10 BMI = 27.8 ± 2.1 kg/m^2^ (SEM) Age not reported	2 g MI + 200 mcg FA daily	200 mcg FA daily	None	Androgenicity: androstenedione, Ferriman-Gallwey score, testosterone Metabolic: fasting insulin, fasting glucose, HOMA-IR Anthropometric: BMI Follow-up: 12 weeks
Iuorno 2002 (35) Venezuela Randomized double-blind RCT	Women with lean PCOS Hospital de Clinicas Caracas in Caracas, Venezuela	DCI = 10 Age = 28.2 ± 1.5 years BMI = 22.4 ± 0.3 kg/m^2^ Placebo = 10 Age = 26.5 ± 1.4 years BMI = 22.1 ± 0.3 kg/m^2^	600 mg DCI daily	Placebo identical daily	None	Androgenicity: total testosterone, free testosterone Metabolic: fasting insulin, fasting glucose, AUC insulin, AUC glucose Lipids and other biomarkers: total cholesterol, triglycerides Anthropometric: BMI Reproductive: ovulation (progesterone >8 ng/mL) Follow-up: 6-8 weeks
Kachhawa 2022 (36) India RCT	Women with PCOS	MI + DCI = 35 Age = 20.8 ± 2.4 years BMI = 24.97 ± 4.03 kg/m^2^ CHC = 35 Age = 20.62± 2.34 years BMI = 24.93 ± 4.09 kg/m^2^	Mychiro (MI 550 mg and DCI 150 mg, USV Private Ltd.) twice a day for 6 months	Monophasic combined hormonal contraceptive pill, (ethinyl estradiol 20 µg and drospirenone 3 mg, Sun Pharmaceutical Industries Ltd)	None	Androgenicity: total testosterone Metabolic: fasting Insulin, fasting glucose, HOMA-IR Anthropometric: BMI Anthropometric: BMI, weight, WHR Reproductive: menstrual cycle length and regularity. Follow-up: 6 months +3 months post-treatment
Khan 2022 (37) Pakistan Parallel prospective cohort study	Teenage girls with PCOS; Department of Obstetrics and Gynaecology, Avicenna Hospital	MI + DCI = 53 Age = 15 ± 3.0 years BMI = 28 ± 4.8 kg/m^2^ Placebo = 53 Age = 15 ± 3.8 years BMI = 28 ± 4 kg/m^2^	MI + DCI (550 mg of MI, 13.8 mg of DCI, 200 µg FA) twice a day	Placebo (FA) 200 µg twice a day	None	Metabolic: fasting glucose Anthropometric: BMI Follow-up: 6 months
Le Donne 2019 (38) Italy RCT (patients were blind to the treatment)	Women with PCOS (Rotterdam) Department of Human Pathology in Adulthood and Childhood “G. Barresi,” University of Messina, Messina, Italy	MI + DCI + FA = 12 Age = 24.1 ± 5.1 years BMI = 31.8 ± 6 kg/m^2^ MI + FA = 10 Age = 25.5 ± 3.4 years BMI = 32.4 ± 5.5 kg/m^2^	MI + DCI + FA; 2 × day Manufactured by Lo.Li.Pharma (Rome, Italy), and each softgel capsule contains 550 mg MI, 13.8 mg DCI, and 200μg FA	MI + FA; 2 × day Manufactured by Lo.Li.Pharma (Rome, Italy), and each sachet contains 2000 mg MI and 200μg FA	Diet (1200 Kcal) administered to all groups according to Italian guidelines, Livelli di Assunzione di Riferimento di Nutrienti 39 and consisted of 25% fats, 15%-18% proteins and the remaining portion glucids; low glycemic index foods were recommended.	Androgenicity: Ferriman-Gallwey score Anthropometric: BMI, waist circumference, hip circumference, WHR, and body composition by bioimpedentiometry Reproductive: menstrual cycle Outcomes assessed at 3 months and 6 months
Leo 2013 (39) Italy Parallel RCT	Women with PCOS (Rotterdam) and insulin resistance (evaluated by HOMA-IR index) Setting not reported (presumed: authors’ affiliation—Obstetrics and Gynaecology Clinic at University of Siena, Italy)	MI + monacolin K = 20 BMI = 28.2 ± 1.3 kg/m^2^ MI = 20 BMI = 28.8 ± 0.7 kg/m^2^ Metformin = 20 BMI = 26.2 ± 0.5 kg/m^2^ Age (total, n = 60) between 24 and 32 years	3 g MI + 6 g monacolin K 3 g (AZELIP-ProgineFarmaceutici), daily for 6 months	Comparison 1: 3 g MI daily for 6 months Comparison 2: 1700 mg metformin, daily for 6 months	Not reported	Androgenicity: FG score, free testosterone, total testosterone, SHBG, Androstenedione Metabolic: fasting glucose, fasting insulin, HOMA Lipids and other biomarkers: total cholesterol, LDL, HDL, triglycerides Anthropometric: BMI Reproductive: menstrual regularity Gastrointestinal adverse events Follow-up: 6 months
Mendoza 2019 (40) Spain Multicenter controlled, randomized, double-blind, parallel group study	Women with PCOS (Rotterdam) undergoing ICSI at 5 clinical sites/centers in Spain	MI + DCI (3.6:1) = 25 Age (total randomized, n = 30) = 31.67 ± 0.86 years BMI (total randomized, n = 30) = 25.51 ± 0.86 kg/m^2^ MI + DCI (40:1) = 19 Age (total randomized, n = 30) = 31.74 ± 0.89 years BMI (total randomized, n = 30) = 24.88 ± 0.69 kg/m^2^	550 mg MI + 150 mg DCI twice daily (3.6:1)	550 mg MI + 13.8 mg DCI twice daily (40:1)	Intake of other vitamins or antioxidants was not permitted during the study except for FA (400 mcg/day), which was provided to all the patients.	Androgenicity: free testosterone Metabolic: fasting glucose, fasting insulin, HOMA-IR Reproductive: pregnancy rate Follow-up: 3 months
Nehra 2017 (41, 42) India Parallel RCT	Women with PCOS (AEPCOS Society 2006 criteria) Age 15-45 Outpatients attending Department of Pharmacology and Obstetrics and Gynecology at Pt BD Sharma PGIMS Rohtak, India	MI = 30 Age = 23.8 ± 0.69 years BMI = 26.45 ± 0.41 kg/m^2^ Metformin = 30 Age = 23.26 ± 1.03 years BMI = 26.09 ± 0.76 kg/m^2^	2 g MI daily	1500 mg Metformin daily	None reported	Androgenicity: testosterone Metabolic: fasting insulin, fasting glucose, glucose/insulin ratio, HOMA-IR Lipids and other biomarkers: total cholesterol, very low-density lipoprotein, LDL, HDL, triglycerides Anthropometric: weight, WHR, waist circumference Follow-up: 24 weeks (outcomes measured at 12 and 24 weeks)
Nestler 1999 (43) Venezuela Parallel double-blind RCT	Women with PCOS Hospital de Clinicas Caracas in Caracas, Venezuela	DCI = 22 Age = 29 ± 6 years BMI = 31.3 ± 2.4 kg/m^2^ Placebo = 22 Age = 26 ± 5 years BMI = 31 ± 2.2 kg/m^2^	1200 mg DCI once daily	Placebo once daily	None	Androgenicity: free testosterone, total testosterone, SHBG, DHEAS, androstenedione Metabolic: fasting glucose, fasting insulin, AUC glucose, AUC insulin Lipids and other biomarkers: total cholesterol, LDL, HDL, triglycerides Anthropometric: BMI, WHR Reproductive: ovulation (progesterone) Follow-up: 8 weeks
Nordio 2012 (44) Italy Parallel RCT	Women with PCOS (Rotterdam) No information about setting (other than Italy)	MI = 24 Age = 28.2 ± 1.5 years BMI = 27.7 ± 2.3 kg/m^2^ MI + DCI = 26 Age = 27.9 ± 1.4 years BMI = 27.5 ± 2.9 kg/m^2^	2 g MI, twice daily	550 mg MI + 13.8 mg DCI twice daily	Patients were asked not to change usual habits for food, sport, and lifestyle.	Androgenicity: total testosterone, free testosterone, DHEAS, SHBG, androstenedione Metabolic: fasting insulin, fasting glucose, HOMA-IR, AUC insulin, AUC glucose Lipids and other biomarkers: total cholesterol, LDL, HDL, triglycerides Anthropometric: BMI, WHR, weight Follow-up: 6 months
Nordio 2019 (45) Italy RCT, open label	Women with PCOS (Rotterdam) University teaching hospital with patients from gynecology/endocrinology clinics, Glasgow, UK	DCI 0:1 = 8 Age = 30.5 ± 2.9 years BMI = 23.48 ± 3.0 kg/m^2^ MI/DCI 1:3.5 = 8 Age = 28.9 ± 3.4 years BMI = 24.52 ± 2.8 kg/m^2^ MI/DCI 2.5:1 = 7 Age = 29.3 ± 3.1 years BMI = 22.87 ± 2.9 kg/m^2^ MI/DCI 5:1 = 8 Age = 31.2 ± 2.7 years BMI = 24.12 ± 2.7 kg/m^2^ MI/DCI 20:1 = 8 Age = 30.6 ± 3.0 years BMI = 23.21 ± 3.1 kg/m^2^ MI/DCI 40:1 = 8 Age = 31.1 ± 3.2 years BMI = 24.08 ± 3.0 kg/m^2^ MI/DCI 80:1 = 8 Age = 29.7 ± 2.8 years BMI = 22.98 ± 2.9 kg/m^2^	2 g MI twice daily at different ratios	None	After the enrollment, the patients were invited to avoid any change of usual habits for food, physical activity, and lifestyle.	Androgenicity: free testosterone, SHBG Metabolic: basal and postprandial insulin levels, HOMA-IR index, Anthropometric: BMI Reproductive: menstruation, ovulation (progesterone), Follow-up: 3 months
Ozay 2017 (46) Turkey Parallel RCT, blinding not specified	Women with PCOS (Rotterdam) and infertility—planning to undergo ovulation induction and IUI University-based OBGYN and fertility clinic in Turkey	MI + FA = 98 Age = 28.65 ± 3.13 years BMI = 24.16 ± 3.11 kg/m^2^ FA alone = 98 Age = 28.81 ± 4.21 years BMI = 25.19 ± 2.41 kg/m^2^	4 g MI + 400 mcg FA daily for 12 weeks prior to FSH/IUI	400 mcg FA daily	FSH + IUI after completing 12 weeks of placebo or intervention	Pregnancy outcomes after FSH + IUI: clinical pregnancy (pregnancy seen on ultrasound with + fetal heart rate), miscarriage rate, number of singleton pregnancies, and number of multiple pregnancies Follow-up: 12 weeks
Pacchiarotti 2016 (47) Italy Double-blind RCT	Women with PCOS (Rotterdam), undergoing first IVF treatment Praxi Pro Vita IVF Centre in Rome, Italy	MI + FA = 166 Age = 31.5 ± 2.8 years BMI = 23.1 ± 1.7 kg/m^2^ FA alone = 195 Age = 32 ± 3.6 years BMI = 22.8 ± 1.3 kg/m^2^ MI + FA + Melatonin = 165 Age = 31.2 ± 2.1 years BMI = 22.8 ± 1.3 kg/m^2^	4000 mg MI + 400 mcg FA. Started treatment first day of IVF cycle and continued until 14 days after embryo transfer	400 mcg FA	IVF	Reproductive: oocyte and embryo quality, clinical pregnancy, implantation rates. Followed until 5 weeks of gestation if pregnant
Papaleo 2009 (48) Italy RCT	Women with PCOS, undergoing ICSI IVF unit, Gynaecologic-Obstetric Department, San Raffaele Hospital, Vita-Salute University, Milan, Italy	MI + FA = 30 Age = 36.2 ± 2.4 years BMI = 26.7 ± 7.5 kg/m^2^ FA alone = 30 Age = 35.4 ± 2.5 years BMI = 26.3 ± 6.8 kg/m^2^	2 g MI twice daily + 400 µg FA	400 µg FA	Controlled ovarian hyperstimulation	Reproductive: pregnancy and implantation rates, live birth and miscarriage rates Not reported (assumed to be up to 12 weeks of gestation)
Pourghasem 2019 (49) Iran Parallel single-blind RCT 3-armed RCT	Women with PCOS (Rotterdam) and letrozole resistance Infertility clinic in University of Medical Sciences in Iran	MI + FA = 50 Age = 31.08 ± 3.31 years BMI = 29.79 ± 3.58 kg/m^2^ Met + FA = 50 Age = 31.06 ± 1.11 years BMI = 27.84 ± 3.68 kg/m^2^ FA alone = 50 Age = 30.42 ± 2.58 years BMI = 27.38 ± 4.02 kg/m^2^	4 g MI + 400 µg FA daily for 3 months	Metformin 1.5 g + 200μg daily 200 µg FA daily for 3 months	Letrozole 7.5 mg daily from third day of menstruation for 5 days in the third cycle	Reproductive: pregnancy rate (primary), livebirth, ovulation Follow-up: 3 months
Raffone 2010 (50) Italy Parallel open-label RCT	Women with PCOS (Rotterdam) Attending IVF Department, G. Martino Hospital, Messina, Italy, for infertility that lasted for a period of more than 14-16 months	MI = 60 Age = 29.1 ± 5.6 years BMI = 25 ± 2.1 kg/m^2^ Metformin = 60 Age = 29.7 ± 6 years BMI = 24.9 ± 2.7 kg/m^2^	Myo-inositol 4 g MI + 400 µg FA daily (for 6 months, if no pregnancy occurred, intervention continued and FSH used for ovulation induction)	1500 mg metformin daily	FSH for ovulation induction if no pregnancy in first 6 months of intervention	Reproductive: spontaneous ovarian activity, pregnancy (biochemical and clinical), and miscarriage rate Adverse events Follow-up: 6 months
Rajasekaran 2022 (51) India Parallel double-blind RCT	Women with PCOS (Rotterdam) undergoing their first IVF cycle Infertility and ART Centre of All India Institute of Medical Sciences, New Delhi, India	MI = 50 Age = 31 ± 3 years BMI = 26 ± 3.1 kg/m^2^ Metformin = 50 Age = 30 ± 4 years BMI = 27 ± 4.2 kg/m^2^	4 g MI daily	1700 mg metformin daily	IVF using a GnRH antagonist protocol after completion of MI or metformin pretreatment × 12 weeks. Medications continued until day of oocyte retrieval	Androgenicity: testosterone, SHBG Metabolic: fasting glucose, postprandial glucose, fasting insulin, HOMA-IR Lipids and other biomarkers: total cholesterol, LDL, HDL Anthropometric: BMI Reproductive: menstrual regularity, clinical pregnancy rate, pregnancy rate per IVF cycle Gastrointestinal adverse events Follow-up: 12 weeks treatment + outcome of IVF cycle until end of pregnancy
Ravn 2022 (52) Denmark Open-label RCT	Women with PCOS (Rotterdam) not attempting pregnancy PCOS outpatient clinic, Department of Gynecology and Obstetrics in collaboration with the Department of Endocrinology, Odense University Hospital, Denmark	MI = 16 Age = 25 (22;34) years (IQR) BMI = 34.2 (30.9;37.2) kg/m^2^ (IQR) Metformin = 12 Age = 27 (24;33) years (IQR) BMI = 35.2 (31.0;39.8) kg/m^2^ (IQR)	2 g MI + 200 mcg FA (Inofolic®, BiO4U Ltd., Dublin, Ireland) twice daily	Metformin: 500 mg (Metformin, Actavis, TEVA, Tel Aviv, Israel) twice daily for two weeks, followed by 1000 mg twice daily	None reported	Androgenicity: FG score, free testosterone, total testosterone, SHBG Metabolic: HOMA-IR, fasting glucose, fasting insulin Lipids and other biomarkers: total cholesterol, LDL, HDL, triglycerides Anthropometric: BMI, waist circumference, weight Reproductive: cycle length Psychological: QoL and depression Adverse events Follow-up: 6 months
Soldat-Stankovic, 2022 (53) Bosnia and Herzegovina Parallel open-label RCT	Women with PCOS (Rotterdam) with irregular periods, infertility, hirsutism, or acne University clinical center, outpatient endocrinology clinic, Banja Luka, Bosnia, and Herzegovina	MI + FA = 33 Nonobese (n = 15) BMI = 21.96 ± 2.34 kg/m^2^ Overweight/obese (n = 15) BMI = 31.03 ± 3.16 kg/m^2^ Metformin = 33 Nonobese (n = 15) BMI = 20.96 ± 2.04 kg/m^2^ Overweight/obese (n = 15) BMI = 30.50 ± 3.52 kg/m^2^ Age (total screened, n = 87) between 18 and 40 years	4 g MI + 400 mcg FA daily	1500 mg metformin daily	None	Androgenicity: hirsutism, free androgen index, total testosterone, SHBG, DHEAS Metabolic: fasting glucose, fasting insulin, HOMA-IR, QUICKI, 120 minutes glucose from OGTT, 120 minutes insulin from OGTT, AUC glucose, AUC insulin Anthropometric: BMI, weight, waist circumference, fat mass Gastrointestinal adverse events Follow-up: 6 months
Tagliaferri 2017 (54) Italy Crossover RCT	Women with PCOS (Rotterdam) Outpatients attending Department of Obstetrics and Gynaecology at Catholic University of Sacred Heart, Rome, Italy	MI = 13 BMI = 31.5 (5.3) kg/m^2^ (IQR) Metformin = 13 BMI = 29.7 (7.8) kg/m^2^ (IQR) Age (total randomized, n = 34) = 25.62 ± 4.7 years BMI (total randomized, n = 34) = 32.55 ±5.67 kg/m^2^	2 g MI daily	1700 mg metformin daily	None reported. Participants advised to continue their usual diet and lifestyle	Androgenicity: FG score, testosterone, free androgen index, DHEAS, SHBG, androstenedione Metabolic: AUC insulin, AUC glucose, peripheral insulin sensitivity Lipids and other biomarkers: total cholesterol, triglyceride, HDL, LDL Anthropometric: BMI, WHR Reproductive: menstrual cyclicity Follow-up: 6 months
Unfer 2011 (55) Italy Parallel RCT	Women with PCOS (Rotterdam) Attending IVF clinic (Department of Obstetrics and Gynecology, University of Messina, Italy) for ovulation induction (and ICSI and without insulin resistance or hyperglycemia)	MI = 43 Age = 35.5 ± 3.2 years BMI = 24.6 ± 8.4 kg/m^2^ DCI = 41 Age = 36.5 ± 2.5 years BMI = 25.3 ± 7.8 kg/m^2^	4 g MI daily for 8 weeks, pre-ICSI	1.2 g DCI daily for 8 weeks, pre-DCI	Controlled ovarian hyperstimulation (GnRH agonist then hCG then recombinant FSH) and ICSI were performed after 8 weeks of MI or DCI	Reproductive: pregnancy (biochemical and clinical) and miscarriage rate Follow-up: 8 weeks plus 1 cycle of ICSI

Abbreviations: AEPCOS, Androgen Excess Polycystic Ovary Syndrome (Society); AMH, anti-Mullerian hormone; AUC, area under the curve; BMI, body mass index; CHC, combined hormonal contraceptive; DCI, D-chiro-inositol; DHEAS, dehydroepiandrosterone sulfate; FA, folic acid; FG, Ferriman-Gallwey; FHR, fetal heart rate; FSH, follicle stimulating hormone; hCG, human chorionic gonadotropin; HDL, high-density lipoprotein; HOMA-IR, Homeostatic Model Assessment for Insulin Resistance; ICSI, intracytoplasmic sperm injection; IQR, interquartile ratio; IUI, intrauterine insemination; IVF, in vitro fertilization; LDL, low-density lipoprotein; LH, luteinizing hormone; mFG, modified Ferriman-Gallwey; MI, myo-inositol; OGTT, oral glucose tolerance test; PCOS, polycystic ovary syndrome; QoL, quality of life; QUICKI, Quantitative Insulin Sensitivity Check Index; RCT, randomized controlled trial; SHBG, sex hormone binding globulin; US, ultrasound; WHR, waist-hip ratio.

### Participants

All participants were individuals with PCOS diagnosed by Rotterdam, National Institutes of Health, or AEPCOS criteria. Mean age ranged from 15 to 36.5, and mean baseline BMI ranged from 20.96 to 32.4 kg/m^2^. Three studies enrolled women with PCOS with an overweight or obese BMI classification (29, 44, 54). Ten studies specifically enrolled women with PCOS who also had infertility (25, 27, 40, 46-51, 55, 56). These studies used co-interventions of fertility treatment, either ovulation induction with letrozole (49), gonadotropin (50), or gonadotropin and intrauterine insemination (46) or in vitro fertilization (IVF) with or without intracytoplasmic sperm injection (ICSI) (25, 27, 40, 47, 48, 51, 55). The fertility co-interventions were either concurrent with the intervention or following a period of pretreatment depending on study design.

### Intervention

Two studies trialed DCI alone (35, 43). Twenty studies compared MI alone at doses of 1 to 4 g daily either alone or in combination with FA 200 to 400 mcg daily (25-27, 30-34, 41, 42, 44, 46-55) against the comparators of metformin, FA alone, or placebo. Three compared the combination of 550 mg MI + 13.8 mg DCI + 200 mcg FA twice daily to FA alone (29, 37, 38). Two studies compared varying ratios of MI + DCI (40, 45).

One trial evaluated MI + DCI + metformin (28) and 1 MI + metformin (30). One had 3 arms comparing MI + monacolin K to MI alone and also to metformin alone (39). We included data only from the MI and metformin arms as the comparison with MI + monacolin K was not considered relevant. One trial evaluated MI + DCI compared to a combined hormonal contraceptive (CHC) (36).

Most trials provided interventions for at least 6 weeks, up to a maximum of 6 months. One study did not clearly define treatment duration (48), and 1 study only provided inositol during IVF stimulation (47). Follow-up ranged from 8 weeks to 6 months and was not reported by 1 study (48).

### Comparator

Three studies were placebo controlled (32, 35, 43). Ten used FA at doses ranging from 200 to 400 mcg daily (25, 27, 29, 31, 34, 37, 46-49). Twelve used metformin at doses of 1000 mg to 2000 mg daily alone or in combination with FA as a comparator (26, 28, 30, 33, 39, 41, 42, 49-54, 56). Three studies used different combinations of MI + DCI as a comparator (40, 44, 45). One study used DCI 1.2 g daily alone as a comparator (55). One study used a CHC, 20 µg ethinyl estradiol and 3 mg drospirenone, as a comparator (36).

### Outcomes

The outcomes reported were heterogeneous. Follow-up duration ranged from completion of an IVF cycle to 6 months after treatment. Up to 23 studies reported on metabolic outcomes including fasting glucose, fasting insulin, HOMA-IR, and quantitative insulin-sensitivity check index, area under the curve (AUC) insulin, and AUC glucose. Twelve studies reported total cholesterol, 10 reported low-density lipoprotein (LDL), high-density lipoprotein (HDL), and triglyceride levels. Twenty-nine studies reported on BMI, 16 reported waist-hip ratio (WHR), 12 reported weight, and 8 reported waist circumference.

Androgenicity outcomes were reported by up to 14 studies and included hirsutism, which was reported as the Ferriman-Gallwey (FG) or modified Ferriman-Gallwey, total and FT levels, sex hormone binding globulin (SHBG), dehydroepiandrosterone-sulphate (DHEAS), androstenedione and free androgen index. Reproductive outcomes were reported by up to 13 studies, specifically menstrual regularity, ovulation rate, pregnancy rates, and live birth rate. Gastrointestinal adverse events (GI AEs) were reported by 6 trials, and other adverse events were reported by 2 trials. One study comparing MI and metformin reported on psychological outcomes (depression and quality of life) (52).

### Risk of Bias

**Figure 2.**
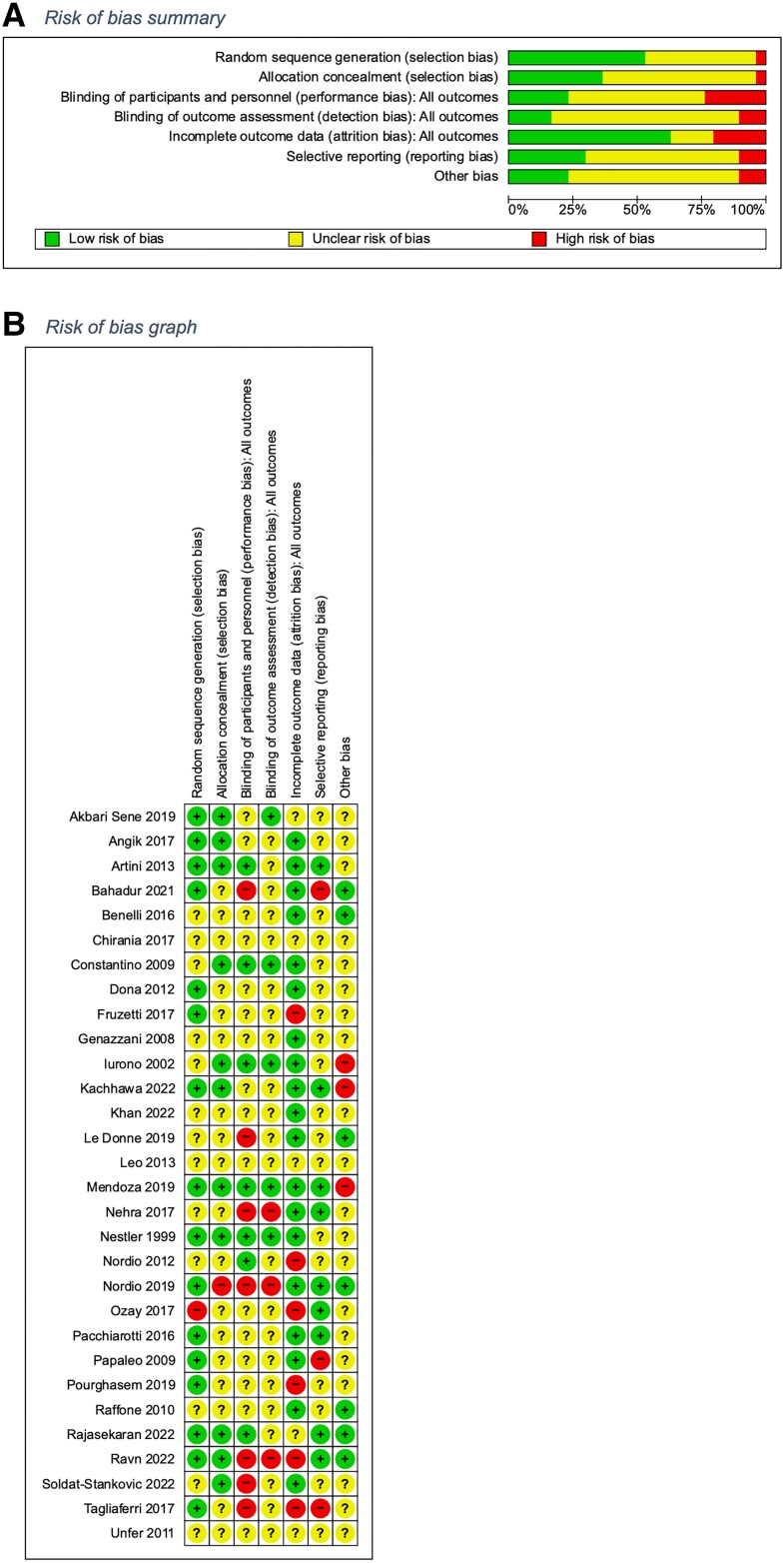
Risk of bias summary and graph.

Figure 2A and 2B describe the risk of bias across studies. Around half to one-third of included trials were judged at low risk of selection bias (16/30 low risk for randomization, 11/30 for allocation concealment). Few trials were judged at low risk of bias for 1 or more of performance (7/30), detection (5/30), reporting (10/30), and other (7/30) bias. Most trials (20/30) were at low risk of attrition bias.

### Effects of Interventions

We report on a total of 13 comparisons. Meta-analyses were conducted on the following 3 comparisons: (1) DCI vs placebo (2 RCTs), (2) MI + FA vs FA (8 RCTs), and (3) MI vs metformin (10 RCTs). For the remaining comparisons, a meta-analysis was not possible on any outcome either due to the comparison only having 1 representative RCT or RCTs reporting nonparametric data (median and interquartile range) or change scores without any information on standard deviation or standard error. For those, we have provided a narrative synthesis.

### Meta-analyses

#### D-Chiro inositol

##### DCI (600-1200 mg/day) v placebo

Two trials reported on this comparison (35, 43). Sample sizes were 20 and 44. The dose of DCI provided ranged from 600 mg (35) to 1.2 g (43) daily. One trial enrolled women with BMI >28 kg/m^2^ (43). There were no co-interventions in either trial. There was very serious imprecision due to very small sample size in both studies; hence all outcomes were of low certainty (see Table 2 for a summary of GRADE assessment for this comparison).

**Table 2.** GRADE table for comparison of DCI vs placebo

Comparison: DCI vs Placebo												
	Quality assessment						No. participants					
No. studies	Design	Risk of bias	Inconsistency	Indirectness	Imprecision	Other	DCI	Placebo	Effect, random [95% CI]	Favors	Certainty	Importance
Outcome: Free testosterone												
2	RCT	No serious risk of bias	No serious inconsistency	No serious indirectness	Very serious*^a^*	None	32	32	MD −0.46 [−0.76, −0.15]	DCI	⨁⨁◯◯ LOW	CRITICAL
Outcome: HOMA-IR												
No studies reported on this outcome.												CRITICAL
Outcome: 2-hour glucose												
No studies reported on this outcome.												CRITICAL
Outcome: BMI												
2	RCT	No serious risk of bias	No serious inconsistency	No serious indirectness	Very serious*^a^*	None	32	32	MD 0.67 [0.10, 1.23]	Placebo	⨁⨁◯◯ LOW	CRITICAL
Outcome: QoL												
No studies reported on this outcome.												CRITICAL
Outcome: Total testosterone												
2	RCT	No serious risk of bias	No serious inconsistency	No serious indirectness	Very serious*^a^*	None	32	32	MD −40.03 [−106.06, 26.01]	Neither (*P* = .23)	⨁⨁◯◯ LOW	IMPORTANT
Outcome: SHBG												
2	RCT	No serious risk of bias	No serious inconsistency	No serious indirectness	Very serious*^a^*	None	32	32	MD 1.79 [0.90, 2.68]	DCI	⨁⨁◯◯ LOW	IMPORTANT
Outcome: DHEAS												
2	RCT	No serious risk of bias	No serious inconsistency	No serious indirectness	Very serious*^a^*	None	32	32	MD −139.43 [−198.51,−80.36]	DCI	⨁⨁◯◯ LOW	IMPORTANT
Outcome: Androstendione												
2	RCT	No serious risk of bias	No serious inconsistency	No serious indirectness	Very serious*^a^*	None	32	32	MD −50.59 [−143.22, 42.03]	Neither (*P* = .28)	⨁⨁◯◯ LOW	IMPORTANT
Outcome: Fasting glucose												
2	RCT	No serious risk of bias	No serious inconsistency	No serious indirectness	Very serious*^a^*	None	32	32	MD −6.14 [−14.52, 2.24]	Neither	⨁⨁◯◯ LOW	IMPORTANT
Outcome: Fasting insulin												
2	RCT	No serious risk of bias	No serious inconsistency	No serious indirectness	Very serious*^a^*	None	32	32	MD −15.53 [−31.10, 0.04]	Neither (*P* = .05)	⨁⨁◯◯ LOW	IMPORTANT
Outcome: AUC glucose												
2	RCT	No serious risk of bias	No serious inconsistency	No serious indirectness	Very serious*^a^*	None	32	32	MD −20.79 [−34.55, −7.10]	DCI	⨁⨁◯◯ LOW	IMPORTANT
Outcome: Total cholesterol												
2	RCT	No serious risk of bias	No serious inconsistency	No serious indirectness	Very serious*^a^*	None	32	32	MD −21.42 [−44.92, 2.09]	Neither (*P* = .07)	⨁⨁◯◯ LOW	IMPORTANT
Outcome: Triglycerides												
2	RCT	No serious risk of bias	No serious inconsistency	No serious indirectness	Very serious*^a^*	None	32	32	MD −31.95 [−63.03, −0.86]	DCI	⨁⨁◯◯ LOW	IMPORTANT
Outcome: WHR												
2	RCT	No serious risk of bias	No serious inconsistency	No serious indirectness	Very serious*^a^*	None	32	32	MD −0.02 [−0.04, 0.01]	Neither	⨁⨁◯◯ LOW	IMPORTANT
Outcome: AUC insulin												
2	RCT	No serious risk of bias	No serious inconsistency	No serious indirectness	Very serious*^a^*	None	32	32	MD −3.65 [−6.65, −0.64]	DCI	⨁⨁◯◯ LOW	IMPORTANT
Outcome: Ovulation rate												
2	RCT	No serious risk of bias	No serious inconsistency	No serious indirectness	Very serious*^a^*	None	32	32	OR 11.50 [3.40, 38.91]	DCI	⨁⨁◯◯ LOW	IMPORTANT

Abbreviations: AUC, area under the curve; BMI, body mass index; CI, confidence interval; DCI, D-chiro-inositol; DHEAS, dehydroepiandrosterone sulfate; HOMA-IR, Homeostatic Model Assessment for Insulin Resistance; MD, mean difference; QoL, quality of life; OR, odds ratio; RCT, randomized controlled trial; SHBG, sex hormone binding globulin; WHR, waist-hip ratio.

^*a*^Downgraded 2 levels due to very serious imprecision: very small sample size (64 participants).

**Figure 3.**
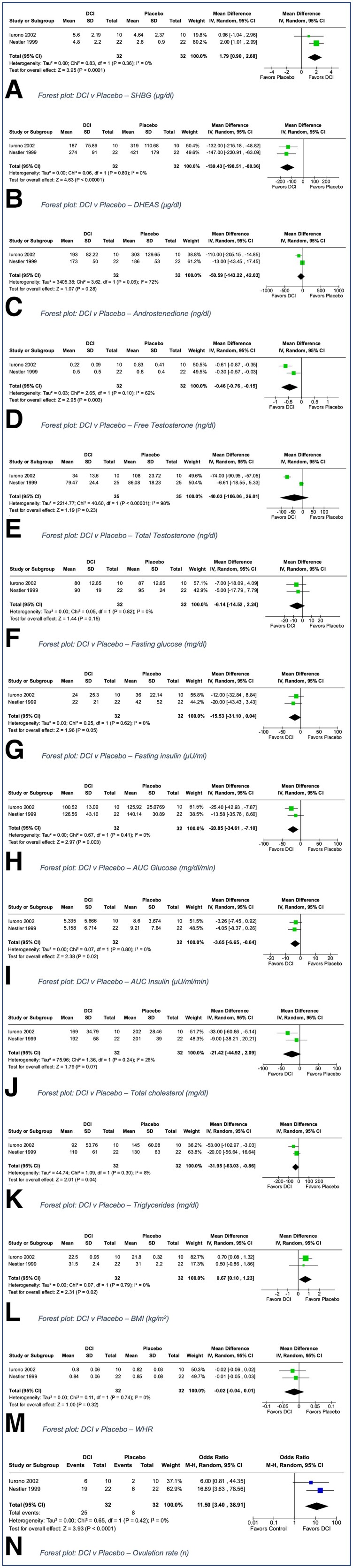
D-chiro-inositol vs placebo forest plots.

Figure 3A–3E shows the forest plots for comparisons regarding androgenicity outcomes. There was low certainty evidence that DCI was superior to placebo for SHBG, DHEAS, and FT (MD = 1.79 mcg/dL, −139.43 mcg/dL and −0.46 ng/dL, respectively; Fig. 3A, 3B, 3D) with no differences in total testosterone or androstenedione (Fig. 3C and 3E). Similarly, DCI was superior to placebo for the metabolic outcomes of AUC insulin (MD −3.65 µU/mL/min, Fig. 3I) and AUC glucose (MD −20.85 mg/dL/min, Fig. 3H), as well as triglycerides (MD −31.95 mg/dL, Fig. 3K), whereas there were no differences for fasting glucose (Fig. 3F), fasting insulin (Fig. 3G), total cholesterol (Fig. 3J), or from single study results for LDL and HDL^43^. Meta-analysis also showed that placebo was superior to DCI for the outcome of BMI (MD 0.67, Fig. 3L) wIth no difference in WHR (Fig. 3m). Ovulation rate was improved with DCI compared with placebo in meta-analysis of 2 trials (OR 11.5; Fig. 3m), both of which counted an ovulatory event if serum progesterone level was >8 ng/mL. Supplemental analysis using SMD for endocrine outcomes were similar to findings from analyses using MD except for FT and AUC glucose, which were no longer statistically significant (17).

#### Myoinositol

##### MI ± FA vs FA

There were 8 studies (25, 27, 31, 34, 46-49) that compared MI + FA to FA alone. Sample sizes in each arm ranged from 10 to 195 per study with a total of 432 participants in the intervention arms and 457 in the control arm across the 8 studies. The dose of MI used was 4 g daily in 6 of the studies (25, 31, 46-49) with 200 to 400 mcg of FA. The other 2 studies evaluated 2 g of MI daily with 200 mcg (34) or 400 mcg (27) of FA. Duration of intervention ranged from 4 to 12 weeks.

Six of the 8 studies enrolled women with PCOS who were presenting for infertility treatment (25, 27, 46-49). MI was provided prior to ovulation induction with letrozole for women with letrozole resistance (40), gonadotropin for ovulation induction and intrauterine insemination (46), IVF (25, 27), and IVF with ICSI (48). One trial randomized women to MI + FA or FA alone during the IVF cycle until 14 days after embryo transfer (47). The remaining 2 studies were not limited to women undergoing infertility treatment (31, 34). One recruited women who were overweight (34) and randomized them to 2 g MI + 200 mcg FA daily or FA 200 mcg daily for 12 weeks. See Table 3 for a summary of GRADE assessments for this comparison.

**Table 3.** GRADE table for comparison of MI + FA vs FA alone

Comparison: MI + FA vs FA												
	Quality assessment						No. participants					
No. studies	Design	Risk of bias	Inconsistency	Indirectness	Imprecision	Other	MI + FA	FA	Effect, random [95% CI]	Favors	Certainty	Importance
Outcome: Free testosterone												
No studies reported on this outcome.												CRITICAL
Outcome: 2-hour glucose												
No studies reported on this outcome.												CRITICAL
Outcome: BMI												
3	RCT	Very serious risk of bias*^a^*	No serious inconsistency	No serious indirectness	Serious imprecision*^b^*	None	58	54	MD 0.14 [−0.49, 0.77]	Neither (*P* = .66)	⨁◯◯◯ VERY LOW	CRITICAL
Outcome: HOMA-IR												
2	RCT	Serious risk of bias*^c^*	No serious inconsistency	No serious indirectness	Very serious*^d^*	None	35	35	MD −1.24[−1.50,−0.99]	MI + FA	⨁◯◯◯ VERY LOW	CRITICAL
Outcome: QoL												
No studies reported on this outcome.												CRITICAL
Outcome: Total testosterone												
3	RCT	Very serious risk of bias*^a^*	No serious inconsistency	No serious indirectness	Serious imprecision*^b^*	None	58	54	MD −25.23 [−78.55, 28.08]	Neither (*P* = .35)	⨁◯◯◯ VERY LOW	IMPORTANT
Outcome: Clinical pregnancy rate												
5	RCT	Very serious risk of bias*^e^*	No serious inconsistency	No serious indirectness	No serious imprecision	None	357	390	OR 1.24 [0.90, 1.73]	Neither (*P* = .18)	⨁⨁◯◯ LOW	IMPORTANT
Outcome: Pregnancy rate (+ hCG)												
3	RCT	Serious risk of bias*^f^*	No serious inconsistency	No serious indirectness	Serious imprecision*^g^*	None	80	80	OR 1.60 [0.84, 3.05]	Neither (*P* = .16)	⨁⨁◯◯ LOW	IMPORTANT
Outcome: Androstenedione												
3	RCT	Very serious risk of bias*^a^*	No serious inconsistency	No serious indirectness	Serious imprecision*^b^*	None	58	54	MD −50.52 [−106.85, 5.81]	Neither (*P* = .08)	⨁◯◯◯ VERY LOW	IMPORTANT
Outcome: Fasting glucose												
2	RCT	No serious risk of bias	No serious inconsistency	No serious indirectness	Very serious*^a^*	None	32	32	MD −6.14 [−14.52, 2.24]	Neither	⨁⨁◯◯ LOW	IMPORTANT
Outcome: Fasting insulin												
2	RCT	Serious risk of bias*^c^*	No serious inconsistency	No serious indirectness	Very serious*^d^*	None	35	35	MD −4.17 [−5.14, −3.20]	MI + FA	⨁◯◯◯ VERY LOW	IMPORTANT

Abbreviations: BMI, body mass index; CI, confidence interval; FA, folic acid; hCG, human chorionic gonadotropin; HOMA-IR, Homeostatic Model Assessment for Insulin Resistance; MI, myo-inositol; MD, mean difference; OR, odds ratio; QoL, quality of life; RCT, randomized controlled trial.

^*a*^Downgraded 2 levels due to very serious risk of bias; 3 studies contribute to this outcome: 1 of the 3 included studies is at unclear risk of bias for most domains, and 1 of the studies is at unclear risk for randomization.

^*b*^Downgraded 1 level for serious imprecision: small sample size (112 participants).

^*c*^Downgraded 1 level due to serious risk of bias: 1 of the 2 included studies is at unclear risk of bias for most domains.

^*d*^Downgraded 2 levels due to very serious imprecision: very small sample size (70 participants).

^*e*^Downgraded 2 levels due to very serious risk of bias; 5 studies contribute to this outcome: 4 have unclear risk of bias in most domains, and 1 has high risk of bias for randomization.

^*f*^Downgraded 1 level for serious risk of bias; 3 studies contribute to this outcome: 1 study has mostly unclear risk of bias across domains, and the other 2 studies have an unclear risk of bias in a blinding domain.

^*g*^Downgraded 1 level for serious imprecision: small sample size for relatively rare events (160 participants).

**Figure 4.**
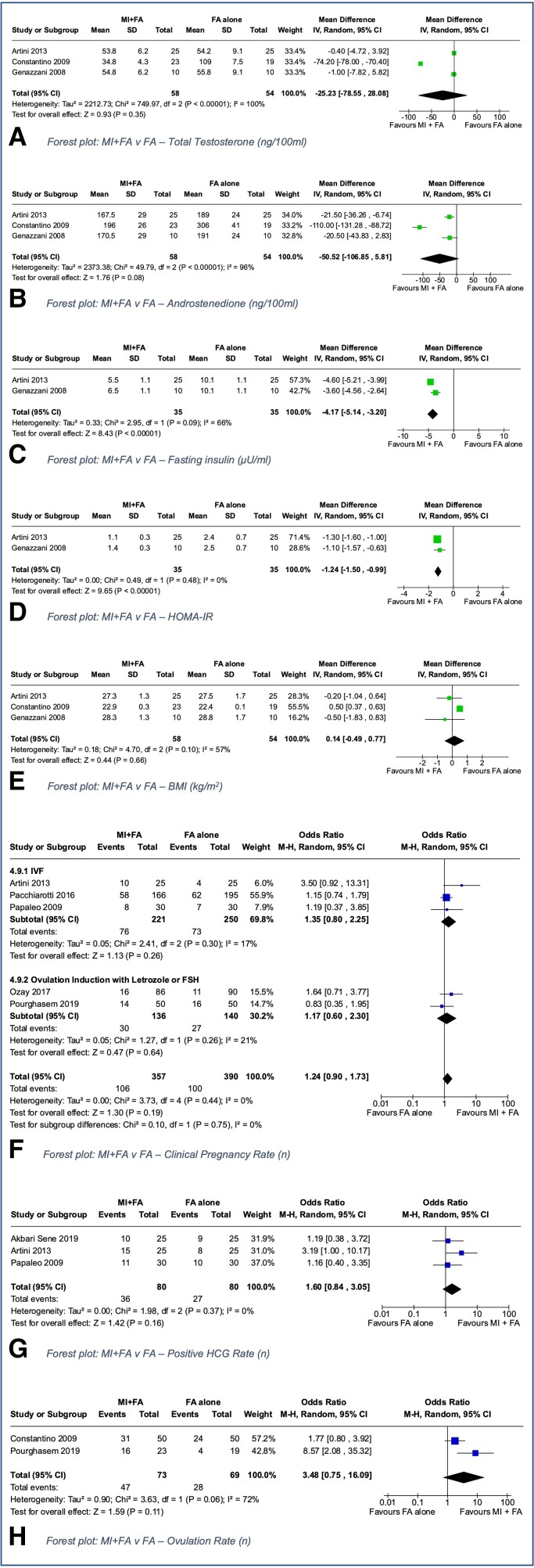
Myo-inositol + folic acid vs folic acid forest plots.

Meta-analysis was conducted on 8 outcomes from 8 studies (25, 27, 31, 34, 46-49). There were no differences between groups for androgenicity outcomes, total testosterone (Fig. 4A, 3 studies), and androstenedione levels (Fig. 4B, 3 studies) (27, 31, 34). For metabolic outcomes, meta-analysis of 2 studies (27, 34) demonstrates that MI + FA was superior to FA alone for fasting insulin (MD −4.17 µU/mL, Fig. 4C) and HOMA-IR (MD −1.24, Fig. 4D), but there was no difference between groups for the outcome of fasting glucose.

There were no differences between groups for other outcomes including BMI (Fig. 4E, 3 studies) (27, 31, 34), clinical pregnancy rate (Fig. 4F, 5 studies) (27, 46-49), rate of positive human chorionic gonadotrophin (Fig. 4G, 3 studies) (25, 27, 48), and ovulation rate (Fig 4H, 2 studies) (31, 49).

Supplemental analysis using SMD for endocrine outcomes was unchanged except for total testosterone and androstenedione where MI + FA was superior to FA (17).

##### MI vs metformin

Ten studies compared MI to metformin and were included in meta-analyses (26, 30, 33, 41, 42, 49-54). Sample sizes in each arm ranged from 12 to 60 per study, with a total of 353 participants in the intervention arms and 356 in the control arm across the 10 studies. The dose of MI ranged from 1 to 4 g, with most studies using 4 g. Doses of metformin ranged from 1000 mg (26, 30) to 2000 mg daily (52). Duration of intervention ranged from 3 to 6 months. Three studies enrolled women with PCOS who were attempting to conceive and utilized co-interventions of letrozole (49), gonadotropin (50), or IVF (51). See Table 4 for the GRADE summary for this comparison.

**Table 4.** GRADE table for comparison for MI vs metformin

Comparison: MI vs Metformin												
	Quality assessment						No. participants					
No. studies	Design	Risk of bias	Inconsistency	Indirectness	Imprecision	Other	MI	Metformin	Effect, random [95% CI]	Favors	Certainty	Importance
Outcome: Free testosterone												
1	RCT	Serious risk of bias*^a^*	NA	NA	Very serious imprecision*^b^*	None	16	12	Unable to calculate	Neither	⨁◯◯◯ VERY LOW	CRITICAL
	
Outcome: 2-hour glucose												
1	RCT	Serious risk of bias*^c^*	NA	NA	Very serious imprecision*^b^*	None	30	30	MD −0.57 (−1.43, 0.29)	Neither (*P* = .20)	⨁◯◯◯ VERY LOW	CRITICAL
Outcome: BMI												
6	RCT	Very serious risk of bias*^d^*	Serious inconsistency*^e^*	No serious indirectness	No serious imprecision	None	210	210	MD 0.03 (−0.63, 0.69)	Neither (*P* = .93)	⨁◯◯◯ VERY LOW	CRITICAL
Outcome: HOMA-IR												
5	RCT	Very serious risk of bias*^f^*	No serious inconsistency	No serious indirectness	No serious imprecision	None	184	182	MD −0.08 [−0.57, 0.41]	Neither (*P* = .74)	⨁⨁◯◯ LOW	CRITICAL
Outcome: QoL												
One study assessed physical functioning, emotional well-being, social functioning, pain, energy/fatigue, and general health (n = 26, high risk of bias). There were no between or within-group differences (very low certainty).												CRITICAL
Outcome: FG score												
2	RCT	Serious risk of bias*^g^*	No serious inconsistency	No serious indirectness	Serious imprecision*^h^*	None	80	80	MD 2.42 [1.52, 3.44]	Metformin	⨁⨁◯◯ LOW	IMPORTANT
Outcome: Total testosterone												
4	RCT	Serious risk of bias*^i^*	No serious inconsistency	No serious indirectness	No serious imprecision	None	160	160	MD 4.69 [−2.55, 11.93]	Neither (*P* = .20)	⨁⨁⨁◯ MODERATE	IMPORTANT
Outcome: SHBG												
2	RCT	Serious risk of bias*^j^*	No serious inconsistency	No serious indirectness	Serious imprecision*^h^*	None	80	80	MD 3.85 [0.50, 7.19]	MI	⨁⨁◯◯ LOW	
Outcome: Clinical pregnancy rate												
4	RCT	Serious risk of bias*^k^*	No serious inconsistency	No serious indirectness	No serious imprecision*^l^*	None	210	210	OR 1.18 [0.79, 1.78]	Neither (*P* = .42)	⨁⨁⨁◯ MODERATE	IMPORTANT
Outcome: Miscarriage rate												
1	RCT	Serious risk of bias	NA	NA	Very serious imprecision	None	29	22	OR 0.89 [0.23, 3.39]	Neither (*P* = .86)	⨁◯◯◯ VERY LOW	IMPORTANT
Outcome: GI side effects												
6	RCT	Serious risk of bias*^m^*	No serious inconsistency	No serious indirectness	No serious imprecision	None	192	190	OR 0.09 [0.02, 0.37]	MI	⨁⨁⨁◯ MODERATE	IMPORTANT
Outcome: Regular menses												
3	RCT	Serious risk of bias*^n^*	No serious inconsistency	No serious indirectness	No serious imprecision	None	125	122	OR 1.85 (0.68, 5.01)	Neither (*P* = .22)	⨁⨁⨁◯ MODERATE	IMPORTANT
Outcome: Ovulation												
2	RCT	Serious risk of bias*^o^*	No serious inconsistency	No serious indirectness	Serious imprecision*^p^*	None	110	110	OR 1.28 [0.59, 2.77]	Neither (*P* = .54)	⨁⨁◯◯ LOW	IMPORTANT
Outcome: Fasting glucose												
4	RCT	Serious risk of bias*^q^*	No serious inconsistency	No serious indirectness	No serious imprecision	None	160	160	MD 0.12 [−2.74, 2.98]	Neither (*P* = .93)	⨁⨁⨁◯ MODERATE	IMPORTANT
Outcome: Fasting insulin												
5	RCT	Serious risk of bias*^r^*	No serious inconsistency	No serious indirectness	No serious imprecision	None	186	188	MD 0.08 [−2.53, 2.53]	Neither (*P* = .95)	⨁⨁◯◯ MODERATE	IMPORTANT
Outcome: LDL												
2	RCT	Serious risk of bias*^s^*	No serious inconsistency	No serious indirectness	Serious imprecision*^t^*	None	80	80	MD 0.19 [−4.44, 4.81]	Neither (*P* = .94)	⨁⨁◯◯ LOW	IMPORTANT
Outcome: Total cholesterol												
2	RCT	Serious risk of bias*^s^*	No serious inconsistency	No serious indirectness	Serious imprecision*^t^*	None	80	80	MD −3.86 [−12.07, 4.34]	Neither (*P* = .36)	⨁⨁◯◯ LOW	IMPORTANT
Outcome: HDL												
2	RCT	Serious risk of bias*^s^*	No serious inconsistency	No serious indirectness	Serious imprecision*^t^*	None	80	80	MD 0.70 [−1.22, 2.62]	Neither (*P* = .47)	⨁⨁◯◯ LOW	IMPORTANT
Outcome: WHR												
2	RCT	Serious risk of bias*^s^*	No serious inconsistency	No serious indirectness	Serious imprecision*^t^*	None	80	80	MD 0.04 [0.01,0.06]	Met	⨁⨁◯◯ LOW	IMPORTANT
Outcome: Weight												
3	RCT	Serious risk of bias*^u^*	No serious inconsistency	No serious indirectness	Serious imprecision*^v^*	None	86	88	MD −0.58 [−4.00, 2.33]	Neither (*P* = .74)	⨁⨁◯◯ LOW	IMPORTANT
Outcome: Waist circumference												
3	RCT	Serious risk of bias*^u^*	No serious inconsistency	No serious indirectness	No serious imprecision	None	110	110	MD 2.52 [−0.89, 5.92]	Neither (*P* = .15)	⨁⨁⨁◯ MODERATE	IMPORTANT

Abbreviations: BMI, body mass index; CI, confidence interval; FG, FG, Ferriman-Gallwey; GI, gastrointestinal; HDL, high-density lipoprotein; HOMA-IR, Homeostatic Model Assessment for Insulin Resistance; LDL, low-density lipoprotein; MD, mean difference; MI, myo-inositol; NA, not applicable; OR, odds ratio; QoL, quality of life; RCT, randomized controlled trial; SHBG, sex hormone binding globulin; WHR, waist-hip ratio.

^*a*^Downgraded 1 level for serious risk of bias: high risk of bias for blinding and attrition.

^*b*^Downgraded 2 levels for very serious imprecision: single study.

^*c*^Downgraded 1 level for serious risk of bias: high risk of bias for blinding, unclear risk of bias for randomization, blinding of outcome assessors, incomplete outcome data, and other bias.

^*d*^Downgraded 2 levels for very serious risk of bias; 6 studies contribute to this outcome: 1 has unclear risk of bias in all domains including randomization and allocation concealment, another 3 studies have an unclear risk in 1 of the blinding domains, 1 study has a high risk of bias in both blinding domains, and 1 study has a high risk of bias for attrition bias. Half of the studies are at unclear risk of bias for either randomization and/or allocation concealment.

^*e*^Downgraded 1 level for serious inconsistency: point estimates vary.

^*f*^Downgraded 2 levels for very serious risk of bias; 5 studies contribute to this outcome: the 1 with the greatest weight has unclear risk of bias for allocation concealment, blinding, selective outcome reporting, and other bias and high risk of bias for attrition; 4 studies are at high or unclear risk of bias for blinding.

^*g*^Downgraded 1 level for serious risk of bias; 2 studies contribute to this outcome: the randomization sequence was unclear in 1 study, blinding was unclear or high risk in both studies, and selective outcome reporting and other bias were unclear in both studies.

^*h*^Downgraded 1 level for serious imprecision: small sample size (160 participants).

^*i*^Downgraded 2 levels for serious risk of bias; 4 studies contribute to this outcome: 2 studies were at unclear risk of bias for randomization, 1 study for allocation concealment; 2 studies were at high risk for blinding, in 2 studies there was unclear risk of bias for selective outcome reporting, and 3 studies were at unclear risk for other bias.

^*j*^Downgraded 1 level for serious risk of bias; 2 studies contribute to this outcome: 1 study was at high risk of bias due to lack of blinding.

^*k*^Downgraded 1 level due to serious risk of bias; 4 studies contribute to this outcome: all have unclear risk of bias in the blinding domain(s), and 1 study is at high risk of attrition bias.

^*l*^Downgraded 1 level due to serious imprecision: small sample size (420) given the relatively rare events of pregnancy.

^*m*^Downgraded 1 level due to serious risk of bias; 6 studies contribute to this outcome: 3 studies are at high risk of bias and 2 are at unclear risk due to blinding of participants; all studies are at high or unclear risk of bias for blinding of outcome assessors; 3 studies are at high risk of bias for attrition.

^*n*^Downgraded 1 level due to serious risk of bias; 3 studies contribute to this outcome: all 3 have unclear risk of bias in 1 or both of the blinding domains.

^*o*^Downgraded 1 level due to serious risk of bias; 2 studies contribute to this outcome: all have unclear risk of bias in one or both of the blinding domains.

^*p*^Downgraded 1 level for serious imprecision: small sample size (220 participants).

^*q*^Downgraded 1 level due to serious risk of bias; 4 studies contribute to this outcome: 2 are unclear risk of bias for randomization, 1 for allocation concealment; 2 are at high risk for blinding in 1 domain and 1 for blinding in 1 domain.

^*r*^Downgraded 1 level due to serious risk of bias; 5 studies contribute to this outcome: 3 are at unclear risk of bias for randomization, 2 for allocation concealment; 2 are at high risk of bias due to blinding in 1 domain and 1 for blinding in another domain.

^*s*^Downgraded 1 level due to serious risk of bias; 2 studies contribute to this outcome: 1 study is at unclear risk of bias for randomization and allocation concealment and at high risk of bias for both blinding domains.

^*t*^Downgraded 1 level due to serious imprecision: small sample size (160 participants).

^*u*^Downgraded 1 level due to serious risk of bias; 3 studies contribute to this outcome: all are at unclear risk of bias for randomization, 2 for allocation concealment; all are at high risk or unclear risk of bias for blinding in both domains.

^*v*^Downgraded 1 level for serious imprecision: small sample size (174 participants).

**Figure 5.**
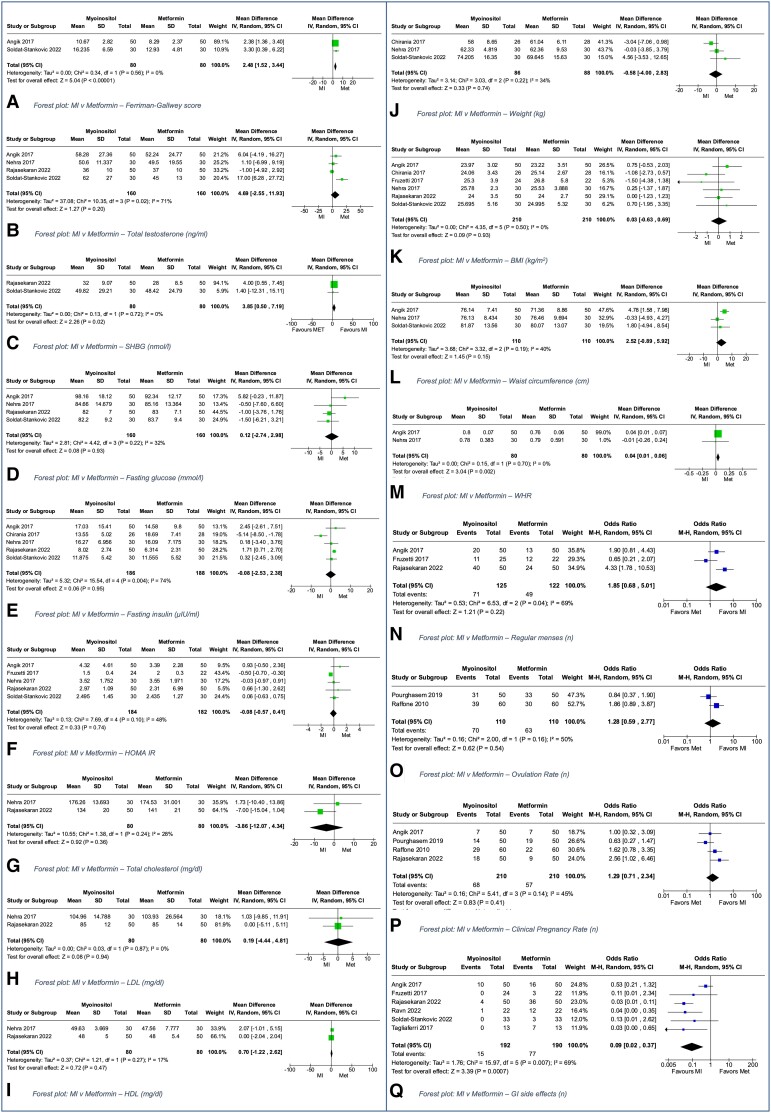
Myo-inositol vs metformin forest plots.

In meta-analysis for androgenicity, metformin was superior to MI for FG score in 2 trials (MD 2.42, Fig. 5A) (26, 53), while MI was superior to metformin for SHBG (MD 3.85, Fig. 5C, 2 studies) (26, 53), with no differences in total testosterone in 4 trials (Fig. 5B) (26, 41, 42, 51, 53). There were no differences between groups for metabolic, lipid, and most anthropometric outcomes including fasting glucose (Fig. 5D, 4 studies) (26, 41, 42, 51, 53), fasting insulin (Fig. 5E, 5 studies) (26, 30, 41, 42, 51, 53), HOMA-IR (Fig. 5F, 5 studies) (26, 33, 41, 42, 51, 53), total cholesterol, HDL, and LDL cholesterol (Fig. 5G–5I, 2 studies) (41, 42, 51), weight (Fig. 5J, 3 studies) (30, 41, 42, 53), BMI (Fig. 5K, 6 studies) (26, 30, 33, 41, 42, 51, 53), or waist circumference (Fig. 5L, 3 studies) (26, 41, 42, 53). However, metformin was superior to MI for the outcome of WHR in analysis of 2 trials (MD 0.04, Fig. 5M) (26, 41, 42).

Reproductive outcomes including resumption of normal menstrual cycles (Fig. 5N, 3 studies) (26, 33, 51) or rate of ovulation (Fig. 5O, 2 studies)(49, 50) did not differ between groups. Four trials (n = 420) (26, 49-51) reported no differences in clinical pregnancy rate, and 1 (n = 120) (50) reported no difference in miscarriage rate between MI and metformin.

In a meta-analysis of 6 trials, GI AEs were less common in the MI group compared with metformin (OR 0.09, 0.02 to 0.37, I^2^ = 69%, 6 trials, Fig. 5Q) (26, 33, 51-54). One study comparing MI and metformin reported on depression and quality of life measures (40). There was no significant difference between the interventions for any of the outcomes measured.

Supplemental analyses using SMD for endocrine outcomes were unchanged except for SHBG where MI was no longer superior to metformin (see Supplementary Data S6). Supplemental analyses adding studies at moderate risk of compromised integrity for critical outcomes of HOMA-IR and BMI still showed no differences between groups for these outcomes (see Supplementary Data S7, Figs. 2 and 3) (17).

##### Sensitivity analysis

Sensitivity analysis including only the 2 trials judged to be at low risk of bias (26, 51) was conducted for the outcomes of clinical pregnancy rate, total testosterone, fasting insulin, fasting glucose, HOMA-IR, BMI, waist circumference, regular menstruation, and GI AEs. See Supplementary Data for more details (17). Metformin was superior to MI for fasting insulin (MD 1.73. 95% CI 0.76-2.71, I^2^ = 0%, 2 trials) while MI was superior to metformin for regular menstruation (OR 2.84, 95% CI 1.26-6.37, I^2^ = 42%), but there was no difference between groups for GI AEs.

### Narrative Syntheses

#### Inositol vs placebo

##### Myoinositol vs placebo

One study (n = 26) (32) comparing 1200 mg MI to matched placebo powder for 12 weeks in women with BMI <25 kg/m^2^ with high risk of bias reported lower body weight (−1.83 kg ± 1.86 vs +0.25 kg ± 0.71, *P* = 0.0055), BMI (−0.69 kg/m^2^ ± 0.69 vs +0.09 kg/m^2^ ± 0.27, *P* = .0058), testosterone (−0.35 nmol/L ± 0.24 vs −0.01 nmol/L ± 0.29, *P* = .0043), androstenedione (−3.96 nmol/L ± 2.16 vs +0.28 mol/L ± 0.39, *P* < .0001), fasting insulin (−2.33 mU/L ± 2.61 vs +1.00 mU/L ± 0.76, *P* = .0018), insulin AUC (−1668.08 mU/L/min ± 1388.52 vs +347.38 mU/L/min ± 314.98, *P* = .0005), and HOMA-IR (−0.54 ± 0.62 vs +0.26 ± 0.16, *P* = .0015) in the MI group vs placebo, respectively. There was very low certainty of evidence for all outcomes.

##### MI + DCI + FA v FA

One study (n = 46) (29) with very low certainty evaluated MI 1.1 g + D chiro inositol 27.6 mg and FA 400μg taken daily compared to FA 400μg alone. No between-group comparisons were conducted. There was no difference between baseline and end of treatment for either arm for the outcomes of DHEAS, androstenedione, fasting glucose, and HOMA-IR. Improvements were reported in the intervention arm for the outcomes of free testosterone, SHBG, and fasting insulin.

##### MI + DCI vs placebo

Another study (n = 106) (37) compared 500 mg MI in combination with 13.8 mg DCI twice daily for 6 months to FA among a population of teenage girls (age 13-19 years) with PCOS. This study had high risk of bias and reported no significant difference in fasting glucose between intervention and placebo group. There was a significant reduction in number of participants with elevated BMI in the intervention group; however, no BMI values or cut offs used for categorization were reported.

#### Inositol vs CHC

##### MI+ DCI vs CHC

One trial (n = 70) (36) assessed outcomes after 6 months of treatment and again 3 months after stopping treatment with MI + DCI compared to CHC. Results suggested no significant difference in weight, BMI, WHR, HOMA-IR, fasting insulin, fasting blood glucose, or testosterone after 6 months of treatment. There was improvement in HOMA-IR and fasting insulin level in the MI + DCI group when assessed 3 months after stopping treatment. Menstrual cycle length was shorter in the CHC group.

#### Inositol + metformin v metformin alone

##### MI + metformin vs metformin

One study (n = 50) (30) showed no difference between groups for BMI, weight, and fasting insulin. Women received either 1 g MI + 1 g metformin daily or 1 g metformin only daily for 4 months.

Supplemental analyses adding studies at moderate risk of compromised integrity for the critical outcome of BMI still showed no differences between groups (see Supplementary Data S7, Fig. 1) (17).

##### MI + DCI + metformin vs metformin

One study (n = 72) (28) suggests there were improvements in menstrual irregularity (38.9% vs 63.9%, *P* = .034), cholesterol (31.58 mg/dL ± 23.99 vs 146.75 mg/dL ± 36.37, *P* = .040), HDL (47.25 mg/dL ± 15.92 vs 41.53 mg/dL ± 6.38, *P* = .049), LDL (85.89 mg/dL ± 19.84 vs 106.16 mg/dL ± 22.78, *P* = .0001), and postprandial insulin (35.30 mIU/L ± 18.94 vs 57.82mIU/L ± 37.52, *P* = .005) with 1.1 g MI + 0.3 g DCI + 1 g metformin vs metformin alone for 6 months, with low certainty of evidence. There were with no differences for hirsutism, androgens, anthropometry, or glucose, with low certainty of evidence.

#### Comparisons between MI and DCI

##### MI vs DCI

One study in euglycemic women undergoing ovulation induction for ICSI (n = 84) (55) with unclear risk of bias suggests that 4 g MI daily for 8 weeks before FSH administration improves total pregnancy rate [22 (51%) vs 10 (24%), *P* < .05] vs 1.2 g DCI daily, but there is no difference for clinical pregnancy, biochemical pregnancy, and miscarriage rates. Evidence is of low certainty.

##### MI + DCI vs MI alone

Two studies (n = 84) reported no differences between MI + DCI and MI alone for critical outcomes (HOMA-IR, BMI, FG score) (40, 44). One study (n = 22) reported no difference for any anthropometric or menstrual outcomes (38). Another study (n = 50) reported improvements seen for free and total testosterone (0.44 ng/dL ± 0.08 vs 0.65 ng/dL ± 0.09, *P* < .05 and 50.4 ng/dL ± 10.2 vs 60.3 ng/dL ± 12.7, *P* < .05, respectively), DHEAS (278 µg/dL ± 32 vs 320 µg/dL ± 31, *P* < .05), fasting glucose (85.9 mg/dL ± 7.2 vs 93.2 mg/dL ± 10.9, *P* < .05), and AUC glucose (12 358 mg/dL/min ± 515 vs 16 209 mg/dL/min ± 447, *P* < .05) but no differences for anthropometric outcomes (including WHR) (44). All outcomes were ranked as very low certainty of evidence. Doses were 1100 mg MI + 27.6 mg DCI (Inofolic Combi) daily v 4 g MI daily for 3 to 6 months with or without 400 mcg FA daily for 12 weeks.

##### MI + DCI vs MI + DCI

Different ratios of MI:DCI were compared in 2 trials (40, 45). In 1 trial (n = 55), 7 ratios were compared: 0:1, 1:3.5, 2.5:1, 5:1, 20:1, 40:1 (standard), and 80:1 for 3 months (45). The total dose of MI + DCI was 4 g daily. In another trial (n = 34), a 3.6:1 ratio was compared with the standard 40:1, also for 3 months (40). There was no difference between groups in either trial for the critical outcome of HOMA-IR. A higher ratio of MI:DCI (40:1 vs 5:1) resulted in lower FT levels (0.55 ng/dL, ± 0.4 vs 1.35 ng/dL, ± 0.7), improved menstrual regularity (5/8, 62.5% vs 1/8, 12.5%), and fasting insulin (16 mU/mL, ± 5 vs 20 mU/mL, ± 13) in 1 trial (45). The evidence was of very low certainty.

##### MI + DCI + FA vs MI + FA

One study (n = 22) (38), with very low certainty, evaluated MI 1.1 g + DCI 27.6 mg and FA 400μg taken daily to MI 4 g + FA 400μg daily after 3 and 6 months. Both arms also included dietary interventions. There was no difference for the outcomes of hirsutism, weight, BMI, WHR, or waist circumference at 3 months or 6 months.

#### GRADE assessments

The majority of outcomes were assessed as being low to very low certainty, as outlined in Tables 2 to 4. Within the comparison of MI vs metformin, the outcomes of total testosterone, clinical pregnancy rate, GI AEs, menstruation, fasting glucose, fasting insulin, and waist circumference were assessed as moderate certainty. The main reasons for downgrading included risk of bias (eg, most studies had a high or moderate risk of bias), inconsistency due to wide CIs or CIs not overlapping, and imprecision (small numbers of studies or sample sizes).

## Discussion

In this systematic review and meta-analysis conducted to inform the 2023 International Evidence-Based PCOS Guideline (15, 57-60), we report on findings from 30 RCTs comparing inositol with various comparators for PCOS. At this time, available evidence regarding the benefits of inositol (in all forms) was inadequate to make evidence-based recommendations regarding efficacy for clinical outcomes. Yet, limited and inconsistent data support some metabolic and hormonal benefits although certainty of the evidence was low to very low. No benefit was found for anthropometric outcomes. While MI resulted in fewer GI AEs compared to metformin, we note that the majority of trials (23/29) did not report on adverse events.

Our findings are consistent with a recent meta-analysis by Jethaliya and colleagues (61) who examined the efficacy of MI against any comparator for anthropometric, metabolic, and endocrine outcomes in PCOS and included 17 RCTs. Similar to our findings, they did not find any improvements in anthropometric (BMI, WHR), metabolic (fasting insulin, fasting glucose, HOMA-IR), or hormonal (luteinizing hormone, FSH, oestradiol, SHBG, DHEAS, and total testosterone levels) outcomes with the exception of androstenedione levels, which favored inositol (61). Our review is more recent than Jethaliya al's, who searched until January 2022, and includes DCI as well as MI. Additionally, we conducted GRADE assessments, which were not performed in Jethaliya et al's review.

Greff and colleagues examined the efficacy of MI or DCI against placebo or metformin in PCOS and reported on 26 RCTs (62). In contrast to our findings, Greff et al (62). reported a higher rate of cycle normalization and greater reduction of BMI and weight in the inositol group compared with placebo. A potential reason for this discrepancy is that Greff and colleagues (62) included some studies that we had excluded from analyses due to uncertainty about research integrity; hence, their results may have been influenced by data that we considered to be questionable in the context of informing guidelines for clinical practice. Moreover, follow-up values were used here, whereas Greff et al used mean change values (delta) between baseline and post-treatment, which tend to produce larger effect sizes and thus more significant results in meta-analyses (63). Ideally, using both estimates in sensitivity analysis would have been the optimal method to ensure robustness of results; however, this was not possible in the present review due to the small number of studies.

Metformin was more efficacious than MI for clinically important outcomes such as central adiposity (WHR) and hirsutism (Ferriman-Gallwey score). Although moderate certainty evidence also suggests that metformin results in a substantially greater number of GI AEs than MI, we note that these adverse events are generally mild and self-limiting (5) and that a sensitivity analysis on studies judged to be at low risk of bias did not detect a difference between groups for GI AEs. Metformin is recommended in the PCOS guidelines for management of anthropometric and metabolic outcomes in women with BMI ≥25 kg/m^2^ and should be used as a first-line treatment. However, it may be reasonable to consider the use of MI as an alternative to metformin in women who cannot tolerate metformin and who require management of anthropometric and metabolic outcomes. It is unclear whether there is any benefit from using a combination of metformin and MI.

Data on the prevalence of use of complementary therapies in PCOS has not been updated for almost 10 years. A 2014 survey of Australian women reported that 70% of respondents used complementary medicines, which were mostly nutritional and herbal supplements (64). In the general population, individuals choose complementary medicines for a number of reasons including feeling unsatisfied with conventional therapy results, alignment with the ideology of holistic health, and for preventive health and well-being, an increased sense of control over one's health, and a perception of safety (65). Similarly, women with PCOS use complementary therapies because they wish to concurrently manage comorbidities such as sleep problems and to improve general well-being (64).

Nevertheless, 70% of women with PCOS cited disadvantages of using complementary medicine such as financial cost and lack of supporting scientific evidence (64). It is important for clinicians to be provided with evidence-based guidance and to understand the motivations for use of complementary therapies and the potential risks, including financial cost, when engaging with patients in informed discussions about use of inositol for PCOS. While complementary medicines are often perceived to be safer than pharmaceuticals, it is important to note that regulation of nutrient supplements varies globally. Most nutritional supplements are not regulated by the European Medicines Agency or US Food and Drug Administration. Studies showing safety and efficacy are generally lacking. In addition, manufacturing standards may vary resulting in inconsistent quality. The cost of inositol is also greater than the cost of metformin. Clinicians should incorporate these considerations together with scientific evidence and individual patient values and preferences when engaging in shared decision-making with their patients about the benefits and risks of using inositol for managing PCOS.

The study has several strengths including the rigorous design and methodology, endorsed by 39 organizations globally as part of the guideline. This topic was highly prioritized by clinicians, patients, and experts who participated in an extensive global prioritization process, and the review was conducted by a multidisciplinary team with substantial expertise in PCOS. Additionally, the validity and trustworthiness of our results is strengthened by the integrity checks incorporated using the RIGID framework, which is now being implemented in 2 further international guidelines. We included GRADE assessments to provide an overview of the certainty of the evidence at the outcome level, in addition to traditional study-level quality appraisal using risk of bias. Limitations of the study are that we only included studies published in English and did not search grey literature. The risk of bias of included studies was generally high, and sample sizes and study numbers were small, which precluded meta-analyses in some instances and decreased the certainty of the evidence overall. Adverse events were not reported by the majority of studies, and there was only 1 study examining adolescents, limiting comparisons in this population subgroup.

## Conclusion

Inositol is extensively used and promoted as effective for management of PCOS and was prioritized by patients internationally in the 2023 international evidence-based PCOS guideline. Yet, this high-quality systematic review and meta-analysis conducted to inform the 2023 international evidence-based guideline on management of PCOS has found that the evidence supporting the use of inositol in the management of PCOS is limited and inconclusive. Some evidence suggests potential benefits of MI or DCI for certain metabolic measures and DCI for ovulation, but inositol may have no effect on other outcomes. The available evidence shows that metformin is superior to MI in improving clinical outcomes such as WHR and FG score. While MI likely causes fewer GI AEs compared with metformin, these GI AEs are typically mild and self-limited.

This meta-analysis, and the guideline, should be used to guide clinical practice and to limit potential harm (including financial harm) to women from using therapies without evidence for effectiveness. Clinicians and patients should consider the uncertainty of the evidence together with individual values and preferences when engaging in shared decision-making regarding the use of inositol for PCOS. Given the consumer interest in using inositol, further research and clinical trials are needed to establish a more robust evidence base and to better understand the potential role of inositol in PCOS management.

## Data Availability

Data are available from the authors upon reasonable request.
