# Correction to: “Inositol for Polycystic Ovary Syndrome: A Systematic Review and Meta-analysis to Inform the 2023 Update of the International Evidence-based PCOS Guidelines”

**DOI:** 10.1210/clinem/dgae588

**Published:** 2024-08-29

In the above-named article by Fitz V, Graca S, Mahalingaiah S, Liu J, Lai L, Butt A, Armour M, Rao V, Naidoo D, Maunder A, Yang G, Vaddiparthi V, Witchel SF, Pena A, Spritzer PM, Li R, Tay C, Mousa A, Teede H, and Ee C (*J Clin Endo Metab*. 2024; 109(6): 1630-1655; doi: 10.1210/clinem/dgad762), there was an error in the References section.

In the originally published article, in the References section, reference 17 read as, “Fitz V, Graca S, Mahalingaiah S, et al. Data from: Inositol for Polycystic Ovary Syndrome: a meta-analysis to inform the 2023 update of the International Evidence-Based PCOS Guidelines. 2023.” In the corrected article, a URL was added to the reference and the reference reads as, “Fitz V, Graca S, Mahalingaiah S, et al. Data from: Inositol for Polycystic Ovary Syndrome: a meta-analysis to inform the 2023 update of the International Evidence-Based PCOS Guidelines. 2023. https://doi.org/10.5281/zenodo.13315672.”

The article has been corrected online.

doi: 10.1210/clinem/dgad762

